# Ultra-Processed Food Consumption during the COVID-19 Pandemic

**DOI:** 10.2174/0118715303373150250310061411

**Published:** 2025-03-17

**Authors:** Domenico Triggiani, Vincenzo Triggiani, Giuseppe Lisco

**Affiliations:** 1 Interdisciplinary Department of Medicine, School of Medicine, University of Bari Aldo Moro, Bari, Italy

## 1 INTRODUCTION

The COVID-19 pandemic affected global dietary habits, amplifying pre-existing trends toward ultra-processed foods (UPF) consumption. Even before the pandemic, an estimated three billion people lacked access to healthy diets, a condition that rose significantly due to the pandemic's economic and social disruptions [1].

UPFs, defined by the NOVA food classification system, are highly processed products designed for affordability, convenience, and extended shelf life. These products, including sweets, sugary drinks, packaged snacks, and ready-to-eat meals, are less expensive, highly palatable, and energy-rich foods often supplemented in fats, sugars, and sodium while low in fiber and essential nutrients [2].

UPF consumption has increased considerably over the last two to three decades at home and away from home [3], and now they account for up to 40% of daily caloric intake in high-income countries and are increasingly consumed in low- and middle-income nations [4] with a strong association between food insecurity and UPF consumption [5]. UPFs have been contributing to a sharp rise in obesity and nutrition-related chronic diseases [6-10], and excessive exposure to UPFs is associated with a higher risk of cognitive impairment and neurodegenerative and cardiovascular diseases [11, 12]. One of the possible links between UPF overexposure and cognitive and cardiovascular risks is probably represented by acrylamide [12, 13].

Apart from some specific reports [14, 15], the pandemic accelerated unhealthy dietary trends toward UPFs [16]. Job losses and income constraints made cheaper and calorie-dense UPFs more accessible than fresh and nutrient-dense foods, especially in moderate and low-income countries. Moreover, social restrictions influenced nutritional behaviors, with most people turning to UPFs as comfort foods to manage stress and depressive mood [17, 18].

As the global prevalence of UPF addiction among the general population is similar to other common addictions, including alcohol and cigarette-smoke addiction (15 to 20%), and raises up to 30% in obese individuals, the existence of a pathophysiological substrate, other than social and economic factors, is highly reasonable. Nevertheless, the precise psycho-neurobiological mechanisms underlying the intricate relationship between food intake, UPF consumption, food addiction, and hexogen stressors are unclear. However, they can involve gut-brain-microbiome dysfunction, psychological complaints associated with cognitive-behavioral and overeating disorders, and binge-type eating [19].

Observational studies confirmed that food addiction and UPF consumption increased remarkably during the COVID-19 pandemic because of psychological distress associated with the pandemic-related constraints, including social isolation and home confinement [18, 20]. However, it is unclear if this behavior can be the cause or the consequence of pandemic-related distress since subsequent studies have confirmed a higher occurrence of anxiety and depression symptoms in patients with a high UPF intake compared to fresh or minimally processed foods [21, 22]. Overall, these results are in line with another observational study that found a 2-to-3-fold increase in the risk of depressive symptoms in patients with high UPF consumption (the highest quartile *vs*. the lowest quartile) and low adherence to the Mediterranean diet (the lowest quartile *vs*. the highest quartile) [23].

Neurobiological studies indicated that intake of sugar- and fat-rich foods is associated with marked activation of several areas of the mesolimbic dopamine pathway, related to food-related gratification, reward, and hedonic eating [24]. However, other pathways can also be involved [25].

Hence, the real need is to characterize the dietary changes that occurred during the pandemic to understand the effects on individual safety and public health.

## 2 INSIGHTS

The paper published by De Nucci *et al.* analyzed 28 studies on UPF consumption before and after the COVID-19 lockdowns. Most studies employed a cross-sectional design, with data predominantly collected through non-probability online surveys targeting European adult populations [26]. Consumption changes were grouped into eight categories: sweets, sugary drinks, snacks, ready-to-eat dishes, processed meats, packaged baked goods, delivery foods, and cereals/energy bars [27].

## 3 INCREASED CONSUMPTION

The lockdown increased the consumption of comfort foods like sweets (*e.g.*, chocolate, pastries) and baked goods (*e.g.*, bread, pizza). Psychological stress during confinement likely fueled this trend, as such foods are known for their serotonin-boosting properties [28]. As another issue, baking and cooking at home became the most popular activities, further driving the consumption of baked goods. Snacks, such as salty or fatty packaged items, also experienced increased demand, often due to more frequent eating patterns during prolonged homestays [29].

Evidence indicates that the consumption of UPFs is strongly associated with an increase in the risk of a wide range of mental and non-mental disorders, including depressive symptoms, anxiety, psychological distress, and depression [30, 31]. Moreover, certain studies have demonstrated a higher consumption of UPFs in patients with hypoplastic alterations in specific cerebral areas within the meso-cortex, including lower volume of the posterior cingulate cortex, left amygdala, ventral putamen, and dorsal-frontal cortex [32]. All these areas have a critical role in regulating appetite and satiety, as well as the approach toward energy-rich and high-palatable foods, food memory, and other crucial functions forming the so-called reward circuit of hedonic eating [33]. Most importantly, the data discussed here are from observational, retrospective studies. Therefore, any supposition about a causative relationship between the two phenomena is actually speculative. Possibly, it can be the contrary. Most people experienced a higher burden of psychological distress during the pandemic, as a massive hexogen stimulus, because of the consequence of background predisposition. So, those at risk of mental health disorders could have shown a higher intake of UPFs than the other.

## 4 DECREASED CONSUMPTION

The consumption of ready-to-eat meals and sugary drinks declined during the lockdown. The availability of more time for cooking and baking at home likely reduced reliance on convenience foods like canned soups and frozen meals. Sugary drinks, often consumed in social settings, could have been more appealing with the cessation of gatherings and outdoor activities [29].

## 5 UNCLEAR TRENDS

There was no clear trend in processed meat consumption during the lockdown period, which may be attributed to supply chain disruptions and increased costs during the pandemic [34]. This stabilization highlights how external economic factors have influenced consumption patterns differently from personal preferences or psychological drivers.

The changes in UPF consumption patterns correlated with observed increases in weight, BMI, and visceral fat during the pandemic. These outcomes elevate the risks of cardiovascular and cerebrovascular diseases, cancers, and frailty. Moreover, higher BMI and visceral fat are associated with more severe COVID-19 outcomes, reinforcing the interconnectedness of dietary habits and pandemic-related health risks [35-37].

## 6 COMMENTARY

The findings highlight the complex interplay between emotional coping mechanisms, economic constraints, and access to food during the pandemic. The increased consumption of UPFs reflects a possible relationship with psychological responses to stress, especially among individuals exposed to an increased risk of mental health disorders.

Preference for comfort foods was also the expression of systemic factors, such as reduced availability and affordability of fresh, nutrient-dense foods, particularly during the lockdowns. On the other hand, the parallel reduction in sugary drinks and ready-to-eat meal consumption was influenced by home confinement, and more time spent in the household possibly increased the time available for home cooking and higher consumption of homemade meals despite the dominance of UPFs.

Any limitation potentially affecting the robustness of the study results should be considered, including the method of data collection, as most information was self-reported from surveys and online questionnaires, the geographical distribution, given that most data were from Europe, and sample size, with most data coming from relatively small-sized cohorts. Overall, limitations may affect the consistency of results.

## CONCLUSION

UPFs induce food addiction; once overexposure to UPFs occurs, it is expected to persist in the absence of specific interventions [38]. Data from Central and South America reveal a flawless association between disruption of daily routine activities and UPF consumption during the pandemic and increasing trends towards overweight and obesity [39-41]. The “day after” (post-pandemic) effect on UPF consumption will clarify future trends in the following years. We are observing a sort of “hungry after the crisis” phenomenon. Food insecurity will worsen as the pandemic has generated or accentuated profound social and economic divergences, especially in middle- and low-income nations. At the same time, mental distress due to pandemic-related health, social, and financial situations may sustain UPF consumption. Both are risk factors for exaggerating UPF consumption, so a further increase in UPF consumption trends is expected [42, 43].

From a public health perspective, therefore, the results of this review emphasize the need for interventions addressing the broader structural determinants of diet. The first level of intervention would include specific dietary education from primary school, teaching children and families to recognize the ingredients of foods and improve cooking skills [44]. The second intervention would include social and occupational policies to ameliorate the management of time dedicated to snakes and food during the daytime, also at school and work, and improve the quality of food canteens. Moreover, policymakers would consider economic policy facilitating the production and commercialization of healthier goods and stricter regulations on UPF marketing, also considering specific taxes on sugar-sweetened foods and beverages to multinational corporations and consumers [45-47]. The last point is important to counteract observed trends indicating that UPF prices decreased considerably during the pandemic while increasing the cost of non-processed or minimally processed foods [48]. Advertising manipulation can be another possible solution to mitigate the commercial appeal of UPFs while sponsoring the consumption of non-processed or minimally processed food, as well as ecologically zero-kilometer food. However, this measure appeared less effective than the others and requires more investigation [45]. Besides educational aspects, labor, social, and economic policies are needed to prevent, treat, and manage food insecurity to effectively counteract UPF consumption in high-risk social categories forced to exaggerate exposure to UPFs because of economic concerns. Moreover, supporting mental health through targeted programs can also mitigate the emotional reliance on high-calorie, low-nutrient foods.

While some changes during the lockdown, such as increased baking, may appear benign, their broader implications for weight gain and metabolic health cannot be ignored in the long term. Future research on more representative populations is needed to understand how socioeconomic and cultural factors intersect with dietary habits during crises. Neurobiological studies are also required to understand better the precise mechanisms underpinning UPF consumption as a coping mechanism or, instead, an eating disorder in the context or separate from a whole and more complex mental disorder or a specific pattern of food addiction [49, 50].

Particular attention should also be paid to emerging and non-emerging metabolic and non-metabolic consequences of UPF consumption, including liver steatosis [51], breast, colorectal, liver, and head and neck cancers [52], gut dysbiosis and related implications in terms of systemic and neuroinflammation [53], and neurodegenerative diseases [54]. Mechanistic studies underlying the relationship between UPF consumption, insulin signaling, neuroinflammation, and carcinogenesis are needed to elucidate the pathophysiological mechanism underlying chronic diseases and develop effective therapeutic strategies to mitigate risks.

Addressing these challenges is vital to fostering resilience in nutritional behaviors and ensuring better public health outcomes.

## ABBREVIATION

UPF = Ultra-Processed Foods
